# Immunopathologic characterization of ultrasound-defined synovitis in rheumatoid arthritis patients in clinical remission

**DOI:** 10.1186/s13075-016-0970-9

**Published:** 2016-03-31

**Authors:** Julio Ramírez, Raquel Celis, Alicia Usategui, Virginia Ruiz-Esquide, Regina Faré, Andrea Cuervo, Raimon Sanmartí, José L. Pablos, Juan D. Cañete

**Affiliations:** Arthritis Unit, Rheumatology Department, Hospital Clinic of Barcelona and IDIBAPS, c/ Villarroel, 170, 08036 Barcelona, Spain; Rheumatology Department, Instituto de Investigación Hospital 12 de Octubre (I + 12), Avda, Córdoba, s/n, 28041 Madrid, Spain

**Keywords:** Rheumatoid arthritis, Remission, Synovitis, Ultrasonography, Immunopathology

## Abstract

**Background:**

Patients with rheumatoid arthritis (RA) in clinical remission may have ultrasound-defined synovitis according to the presence of power Doppler (PD) signal. The objective was to describe the immunopathologic characteristics of ultrasound-defined synovitis compared with synovitis in patients with clinically active RA.

**Methods:**

We included between 6 and 8 ultrasound-guided synovial biopsies per patient from 20 patients with RA in clinical remission (DAS28-ESR <2.6) with PD signal, 22 synovial tissue samples (ST) from patients with clinically active RA (swollen joint with confirmed inflammatory synovial fluid) as inflammatory controls, and 10 ST from non-inflammatory controls. Immunostaining for CD3 (T lymphocytes), CD20 (B lymphocytes), CD68 (macrophages), CD117 (mast cells), hsp47 (fibroblasts), bFGF and CXCL12 (angiogenic factors) was made and quantified by digital image analysis. The number of CD31 vessels/mm^2^ was quantified.

**Results:**

RA patients in remission with PD signal had significantly reduced synovial T-cell, B-cell, mast cell and fibroblast density, but similar macrophage infiltration compared with patients with clinically active RA. Vascularity, bFGF and CXCL12 were partially reduced in RA patients in remission with PD signal compared to those with active RA, but were significantly higher compared with ST from non-inflammatory controls. During the 12-month follow up, 8/20 RA patients (40 %) lost remission: all had synovial hypertrophy grade ≥2 and significantly more synovial B cells and mast cells than patients maintaining remission.

**Conclusions:**

Asymptomatic ultrasound-defined synovitis and clinically active arthritis differ in the degree of infiltrating lymphoid, mast cells and fibroblast density, but are similar with respect to macrophage infiltration. Persistently increased angiogenic factor expression and vascularity may explain the persistence of a PD signal.

## Background

Early treatment, the availability of biologic therapies and treat-to-target strategies has made remission a realistic goal in rheumatoid arthritis (RA) [1–5]. However, significant numbers of RA patients classified as being in clinical remission have detectable synovitis on ultrasound (US) or magnetic resonance imaging (MRI) [6–10]. These patients have an increased probability of reactivation of RA and/or radiological progression during follow up. The persistence of subclinical synovitis, as evaluated by power Doppler (PD) US has been associated with a high risk of flares and joint damage [11–14]. Understanding the immunopathology underlying US-defined synovitis in RA patients in remission may increase knowledge of the physiopathology of RA and identify potential biological biomarkers of further reactivation and the progression of structural damage.

Studies suggest that PD scores correlate with vascular and inflammatory cell infiltration [15, 16]. However, in RA patients in clinical remission, only retrospective, limited semiquantitative pathological data obtained from the joints of patients undergoing replacement surgery have been reported [17]. To identify the pathological basis of these findings, we aimed to analyze the immunopathologic characteristics of synovial tissue (ST) obtained from RA patients in clinical remission with US- defined synovitis (presence of PD signal) and to determine whether immunopathologic changes predict the relapse from clinical remission during a 12 months of follow up.

## Methods

### Patient recruitment

Consecutive patients with RA were selected, who had been in clinical remission for ≥6 months and had PD signal. Patients had a 28-joint Disease Activity Score (DAS28) <2.6 and no swollen or tender joints as evaluated by two independent rheumatologists in the RA outpatient clinic. Patients aged <18 years, with allergy to local anesthetics, anticoagulant treatment, or unhealthy skin at the site of interest, were excluded. Clinical and US examinations were performed at the outpatient clinic of the rheumatology service and the US-guided synovial biopsy was carried out within 24 h of the clinical and US examination. We obtained biopsies from 24 patients, but 4 of the biopsies were non-evaluable due to a lack of a well-defined synovial lining. Finally, 20 patients with suitable synovial samples were clinically followed for 12 months to detect relapse from remission. All 20 patients completed the follow up.

We also included ST from two control groups: (1) synovial biopsies from unselected patients with clinically active RA (n = 22), and (2) non-inflammatory ST (control) obtained on arthroscopic surgery from 10 selected patients (60 % male; mean age (± SD) 42 (±8) years), who had meniscal lesions, but had no osteoarthritic or inflammatory lesions on MRI and had histologically normal ST. The study was approved by the Ethics Committee of the Hospital Clinic of Barcelona (Comité Ético de Investigación Científica del Hospital Clínic de Barcelona, Spain (2011/6490)) and signed informed consent was obtained from each patient.

### US assessment and US-guided synovial biopsy

All US assessments were performed using high-sensitivity US equipment (Acuson Antares®, Siemens AG, Erlangen, Germany), using a frequency range from 10 to 12 MHz and pulse repetition frequency between 500 and 800 Hz. Receiver gain settings were controlled to eliminate the appearance of artifacts. Joint US findings were characterized according to published Outcome Measures in Rheumatology (OMERACT) definitions [18]. An experienced sonographer (JR), blinded to the results of the clinical joint examination, evaluated both knees and 11 joints in each hand (including proximal interphalangeal joints, metacarpophalangeal joints (MCP) and wrists) for synovial hypertrophy (SH) and intra-articular PD signal according to European League Against Rheumatism (EULAR) guidelines [19]. SH and PD scores were independently quantified (grades 0–3). US-defined synovitis was based on the presence of PD signal. Intra-rater agreement was calculated as previously described [20], and was 0.81 for SH and 0.92 for PD.

US-guided synovial biopsies were performed using the same US equipment. Two operators (JDC and JR) performed all biopsies. Only joints with PD signal were selected for biopsy. Biopsies were carried out in an operating theatre and six to eight synovial biopsies were taken per procedure. Each US biopsy was made according to the technique described by Kelly et al. [21]. There were no complications after the procedures.

### Histological and immunohistochemical assessments

ST were routinely fixed and embedded in paraffin. Deparaffinized sections were cooked to perform antigen retrieval when required. Slides were subsequently stained with an automated immunostainer (TechMate 500 Plus; Dako, Cambridge, UK) using the following monoclonal antibodies: anti-CD3 (clone PS1; Novocastra, Newcastle, UK) for T-lymphocytes, anti-CD20 (clone L26; Dako) for B-lymphocytes, anti-CD68 (clone KP-1; Dako) for CD68 + macrophages, anti-CD117 (rabbit anti-human polyclonal antibody; Dako) for mast cells, anti-Hsp47 monoclonal antibodies (IgG2b M16.10A1 clone; Assay Designs) for synovial fibroblasts, anti-CD31 (clone JC70A; Dako) for endothelial cells, and anti-basic fibroblast growth factor (bFGF) (polyclonal SC-79, Santa Cruz Biotechnology, Santa Cruz, CA, USA) and anti-CXCL12 (clone K15C [22]) for angiogenic markers, which were significantly increased in serum from these patients in our previous study [20]. As a negative control, the primary antibodies were substituted by isotype-matched and concentration-matched control antibodies. The primary antibodies were subsequently detected by an avidin-biotin-peroxidase-based method (Envision System; Dako) and an aminoethylcarbazole color reaction (Sigma-Aldrich, St. Louis, MO, USA) as previously described [23]. Finally, the slides were counterstained with hematoxylin.

### Digital image analysis

Stained slides were scored on digital image analysis by an independent observer (RC), who was blinded to the diagnosis and the clinical data. Only slides with well-defined lining and sub-lining areas were included. Each stained slide was scored in its entirety by dividing it into different regions. Within each region, the number of stained cells per area and the percentage of stained cells were measured in at least 20 high-power fields using the AnalySIS®Imaging processing program (Olympus®) as previously described [24].

### Statistical analysis

Quantitative immunohistologic data were analyzed using the nonparametric Mann–Whitney test or the Kruskal-Wallis test with the post-hoc Dunn test where appropriate. Correlation between variables was analyzed using Spearman’s test. Values of *p* < 0.05 were considered significant. The statistical analyses were carried out using SPSS V.18 software.

## Results

### Clinical, demographic and US data

ST samples from 20 patients with RA in remission, who had a PD signal, 22 ST samples from RA patients with active synovitis (swollen and tender joint with inflammatory synovial fluid), and 10 ST samples from non-inflammatory controls were included. The clinical and demographic data for the RA patients are shown in Table 1. In both the active RA and remission RA groups, the joint with greatest disease activity as assessed by US was selected for biopsy. This means there was the highest grade of PD signal and the highest grade of SH in joints with a similar grade of PD signal. Most biopsies in the remission group were taken from the wrist and MCP, the joints that were involved more frequently. Biopsies in the active group were mostly taken from the knee due to the additional therapeutic effect of the arthroscopic lavage.

**Table 1:** Clinical and demographic data for patients with rheumatoid arthritis (RA)

	Active RA (n = 22)	Remission RA (n = 20)	*P* value
Female, *n* (%)	16 (72.7)	15 (75)	0.867
Age, years, mean (SD)	58.8 (9.6)	53.7 (10.8)	0.346
Disease duration, years, mean (SD)	12.5 (9.8)	8.5 (8.2)	0.001
Rheumatoid factor^+^, *n* (%)	14 (63.6)	11 (55)	0.569
ACPA^+^, *n* (%)	15 (68.1)	18 (90)	0.085
DAS28-ESR, mean (SD)	5.41 (1.30)	1.92 (0.32)	0.001
Biological therapy, *n* (%)	14 (63.6)	10 (50)	0.231
Prednisone, *n* (%)	18 (81.8)	2 (10)	0.0001
DMARDs, *n* (%)	20 (90.9)	16 (80)	0.881
Biopsy location			
MCP, *n* (%)	0 (0)	3(15)	
Wrist, *n* (%)	4 (18.1)	13 (65)	
Knee, *n* (%)	18 (81.8)	4 (20)	
PD, *n* (%)	22 (100)	20 (100)	
PD ≥2, *n* (%)	22(100)	3 (15)	
SH ≥2, *n* (%)	22 (100)	16 (80)	
SH ≥2 + PD, *n* (%)	22 (100)	16 (80)	

*SD* standard deviation, *ACPA* anti-cyclic citrullinated peptide/protein antibody, *DAS28* 28-joint Disease Activity Score, *ESR* erythrocyte sedimentation rate, *DMARD* disease-modifying antirheumatic drug, *MCP* metacarpophalangeal joint, *PD* power Doppler, *SH* synovial hypertrophy

Patients with active disease had longer disease duration and, as expected, higher DAS28 and greater use of prednisone compared with patients in remission, who had a PD signal (Table 1). No significant differences in the percentages patients on DMARDs or biologic therapy were found between the active RA and remission RA groups. The duration of clinical remission was 37 (8–58) months (median (IQR)).

Of the 20 patients in remission, who had PD signal, 16 (80 %) also had SH grade ≥2, fulfilling a previously reported more stringent criterion of synovitis (SH grade ≥ grade 2 plus a PD signal). This definition is based on the concept that synovitis is the presence of synovial villae (hypertrophy) with active vessels (PD signal) and identifies a subgroup of RA patients in clinical remission with significantly greater disease activity and higher serum levels of angiogenic cytokines [20]. All RA patients with clinically active disease had SH grade ≥2 and a moderate-to-severe signal (PD ≥2), whereas 85 % of RA patients in clinical remission had a mild PD signal (PD = 1) and 15 % had a PD signal = 2 (Table 1).

### Immunopathologic characterization of RA patients in clinical remission

**Fig. 1:**
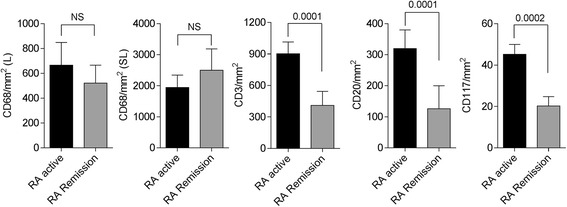
Immunohistochemical analysis of inflammatory infiltrating cell populations in synovial tissues of rheumatoid arthritis (*RA*) patients in remission, who had power Doppler signal, compared to patients with clinically active RA. The density of CD68 (macrophages) in the synovial lining (*L*) and sub-lining (*SL*), CD3 (T lymphocytes), CD20 (B lymphocytes) and CD117 (mast cells), as assessed by quantitative digital image analysis is shown. Data are represented as mean ± SD. *NS* not significant

We first analyzed potential differences in the density of inflammatory cell infiltration between ST from RA patients in remission, who had PD signal, and patients with clinically active RA. ST from patients in remission, who had PD signal, had significantly reduced density of CD3^+^ T lymphocytes (*p* = 0.0001), CD20^+^ B lymphocytes (*p* = 0.0001) and CD117^+^ mast cells (*p* = 0.0002) compared with patients with clinically active RA. No significant differences in the density of total, lining, or sub-lining CD68^+^ macrophages were found between patients with clinically active RA and those in remission (Fig. 1).

**Fig. 2:**
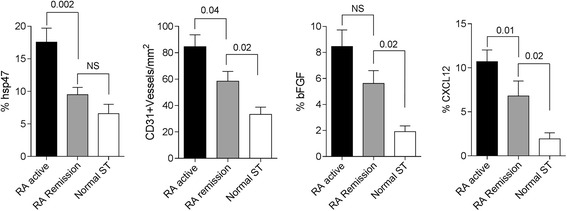
Immunohistochemical analysis of stromal and vascular cells, and expression of angiogenic factors in synovial tissues (*ST*) from rheumatoid arthritis (*RA*) patients and non-inflammatory synovial tissues from controls (*Normal ST*). The density of of hsp47 (fibroblasts), CD31 (blood vessels), and the expression of basic fibroblast growth factor (*bFGF*) and CXCL12, as assessed by quantitative digital image analysis is shown. Data are represented as mean ± SD. *NS* not significant

As vascular and stromal changes have been suggested to explain the persistence of subclinical inflammation, we analyzed these changes in ST from the three study groups. Patients in remission, who had a PD signal, had significantly reduced density of CD31^+^ blood vessels compared with patients with active RA (*p* = 0.04), but had significantly increased density compared with non-inflammatory ST samples from controls (*p* = 0.02). The density of hsp47^+^ fibroblastic cells was significantly reduced in RA patients in remission who had a PD signal compared to patients with active RA (*p* = 0.002) and was similar to that in controls with no inflammatory disease (Fig. 2).

We also analyzed the expression of bFGF and CXC12, two angiogenic factors that we previously found to be increased in serum from RA patients in remission, who had PD signal, [20]. The expression of CXCL12 was significantly increased in patients with clinically active RA compared with those in remission, but CXCL12 expression in the remission group was still significantly higher than in the control group who did not have inflammatory disease (Fig. 2). In contrast, the expression of bFGF in the remission group did not significantly differ from that in the group clinically active RA, but was significantly higher than in the non-inflammatory control group (Fig. 2).

### High risk of flare in patients with RA in remission who had power Doppler signal and synovial hypertrophy ≥2

All patients in remission, who had PD signal, were followed for 12 months, during which 8 (40 %) came out of remission, all of whom met a more stringent criterion for US synovitis (PD signal plus SH grade ≥2) on biopsy. These patients had a significantly higher density of CD20+ B cells (*p* = 0.009), CD117^+^ mast cells (*p* = 0.010), and a non-significant trend towards higher density of lining macrophages (*p* = 0.079) than patients maintaining clinical remission (Table 2).

**Table 2:** Immunopathologic features of patients with rheumatoid arthritis who had come out of remission at 12 months

	Remission (n = 12)	Out of remission (n = 8)	*P* value
CD3/mm^2^	215.3 (258.1)	622.6 (1038.9)	0.232
CD20/mm^2^	25.7 (17.2)	276.3 (506.0)	0.009
CD31/mm^2^	53.8 (28.4)	54.6 (27.7)	0.953
CD68 total/mm^2^	1793.2 (2049.2)	4833.5 (4149.5)	0.329
CD68 lining/mm^2^	166.7 (254.7)	905.4 (877.4)	0.079
CD68 sub-lining/mm^2^	1500.8 (2036.2)	3927.9 (3343.9)	0.205
CD117/mm^2^	10.1 (8.9)	43.1 (27.6)	0.010
bFGF, %	4.5 (3.6)	7.2 (5.2)	0.217
Hsp47, %	91.1 (285.9)	134.4 (349.3)	0.247

Data are expressed as mean (SD). *bFGF* basic fibroblast growth factor, *Hsp47* heat shock protein 47

## Discussion

To our knowledge, this is the first study to quantitatively analyze changes in the infiltrating and resident cell components that characterize RA patients in remission with US-defined synovitis, based on the presence of PD signal. Previous studies in active or end-stage joints in patients with RA and osteoarthritis have shown correlation between US findings, mainly PD scores, and vascularity [15]. In a recent study of patients with clinically active RA there was significant correlation between inflammatory cell infiltration (CD68 macrophages and CD3 T lymphocytes) and vascularity with a PD color fraction [16].

Our results show that RA patients in clinical remission, who have a persistent PD signal, have high macrophage infiltration, comparable to patients with clinically active RA, and increased vascularity. In contrast, lymphocytic and mast cell infiltration and fibroblastic hyperplasia were significantly reduced in these patients compared with patients with clinically active disease. The comparison with ST from individuals without inflammatory disease reinforces these conclusions as there was increased vascularity but similar fibroblastic density in RA patients in remission compared with synovium from patients without inflammatory disease. Furthermore, the expression of angiogenic factors, particularly bFGF, which was found to be the best systemic biomarker of US synovitis in our previous study [20], was also significantly elevated in the ST of these patients.

These results have implications for the mechanistic understanding of US-defined synovitis in RA patients in clinical remission. Synovial macrophage infiltration is the best marker of active disease, and the most sensitive to change after effective therapy [25, 26]. Therefore, the finding of persistent macrophage infiltration suggests that US synovitis does not differ physiopathologically from clinically active synovitis. Despite the absence of clinical signs (i.e., non-tender and non-swollen joints), RA patients in remission, who have PD signal, would appear to have a pathologic status whereby macrophage depletion has not been achieved by therapy.

Our results also confirm the link between PD and increased vascularity in these patients, as was previously suggested to be characteristic of joints in clinically active disease [15, 16]. The persistent expression of angiogenic bFGF and macrophage infiltration provides a mechanistic explanation for this pathologic observation. Interestingly, bFGF-induced angiogenesis has been linked to macrophage infiltration, underlining the pathogenic role of this factor in RA [27, 28].

The main pathologic difference between clinical and US-defined synovitis was in lymphocytes, mast cells and fibroblasts, which were significantly-reduced in ST from RA patients in remission who had PD signal. These cells might, therefore, play a relevant role in the clinical expression of synovitis, but identification of the potential mechanisms remains speculative. It is also interesting that the levels of mast cell and B lymphocyte infiltration were associated with clinical progression to symptomatic, clinically active RA during the follow up.

Mast cells play an essential role in some animal models of arthritis [29] and are an important synovial reservoir of pro-inflammatory cytokines, including TNFα and IL-17, in RA [30, 31]. However, few studies have involved analysis of their changes or their prognostic implications in patients on treatment [32]. Despite the significant reduction in asymptomatic US synovitis compared with clinical synovitis, the levels in patients in remission correlated with further clinical reactivation during follow up, supporting a role for mast-cells in disease activity and in the transition from subclinical to clinically active synovitis.

A higher density of synovial B lymphocytes was also associated with RA reactivation. The efficacy of anti-CD20 therapy in RA has shown the pathogenic relevance of B cells, but the specific role of local synovial B-cells is less clear [33]. An increased basal density of B cells has been associated with a worse therapeutic response to anti-CD20 but not to other therapies [34, 35].

Our study was limited by the relatively small sample size. However, we were able to take synovial biopsies from >80 % of eligible patients (meeting the PD signal criteria). However, ST from the group with clinically active RA was taken from joints with clinically active disease (swollen joints with inflammatory synovial fluid) and was included retrospectively to specifically compare their immunopathologic characteristics with the RA remission group. Therefore, the association between the immunopathologic findings and disease reactivation are only exploratory and require further confirmation. Finally, 80 % of patients with RA in remission, who had PD signal, also had synovial hypertrophy grade ≥2, thus, meeting a more stringent criterion for US synovitis [20]. These patients had a high risk of coming out of remission, as 50 % had arthritic flares during follow up.

## Conclusions

This prospective study shows that the presence of US-defined synovitis based on the presence of PD signal in RA patients in clinical remission has a histopathological correlate, which is characterized by an unbalanced reduction in the cellular components of clinically active RA synovitis, with persistent macrophage and vascular components. These results, together with our previous observations on serum biomarkers [20], support a specific cytokine profile in these patients, with the angiogenic factor bFGF emerging as a potentially relevant mediator.

## Abbreviations

CXCL: CC-chemokine ligand
DAS28: 28-joint Disease Activity Score
ESR: erythrocyte sedimentation rate
bFGF: basic fibroblast growth factor
hsp: heat shock protein
IL: interleukin
IQR: interquartile range
MCP: metacarpophalangeal joints
MRI: magnetic resonance imaging
PD: power Doppler
RA: rheumatoid arthritis
SD: standard deviation
SH: synovial hypertrophy
ST: synovial tissue
TNF-α: tumor necrosis factor-α
US: ultrasound
